# Determining rib fracture age from CT scans with a radiomics-based combined model: a multicenter retrospective study

**DOI:** 10.1186/s13244-023-01546-y

**Published:** 2023-12-10

**Authors:** Yilin Tang, Liang Jin, Wenbin Ji, Zhuangxuan Ma, Dechun Li, Wei Hong, Ming Li

**Affiliations:** 1 Radiology Department, Huadong Hospital Affiliated to Fudan University, No. 221 West Yan’an Road, Jing’an District Shanghai, 200040 China; 2 Radiology Department, Huashan Hospital Affiliated to Fudan University, Shanghai, China; 3 Radiology Department, Shanghai Electric Power Hospital, Shanghai, China; 4 Department of Geriatrics and Gerontology, Huadong Hospital Affiliated to Fudan University, No. 221 West Yan’an Road, Jing’an District Shanghai, 200040 China; 5 Institute of Functional and Molecular Medical Imaging, No. 221 West Yan’an Road, Jing’an District Shanghai, 200040 China

**Keywords:** Radiomics, Rib fracture, Computed tomography, Machine learning

## Abstract

**Objectives:**

We aimed to develop a combined model based on clinical and radiomic features to classify fracture age.

**Methods:**

We included 1219 rib fractures from 239 patients from our center between March 2016 and September 2022. We created an external dataset using 120 rib fractures from 32 patients from another center between October 2019 and August 2023. According to tasks (fracture age between < 3 and ≥ 3 weeks, 3–12, and > 12 weeks), the internal dataset was randomly divided into training and internal test sets. A radiomic model was built using radiomic features. A combined model was constructed using clinical features and radiomic signatures by multivariate logistic regression, visualized as a nomogram. Internal and external test sets were used to validate model performance.

**Results:**

For classifying fracture age between < 3 and ≥ 3 weeks, the combined model had higher areas under the curve (AUCs) than the radiomic model in the training set (0.915 vs 0.900, *p* = 0.009), internal test (0.897 vs 0.854, *p* < 0.001), and external test sets (0.881 vs 0.811, *p* = 0.003). For classifying fracture age between 3–12 and > 12 weeks, the combined model had higher AUCs than the radiomic model in the training model (0.848 vs 0.837, *p* = 0.12) and internal test sets (0.818 vs 0.793, *p* < 0.003). In the external test set, the AUC of the nomogram-assisted radiologist was 0.966.

**Conclusion:**

The combined radiomic and clinical model showed good performance and has the potential to assist in the classification of rib fracture age. This will be beneficial for clinical practice and forensic decision-making.

**Critical relevance statement:**

This study describes the development of a combined radiomic and clinical model with good performance in the classification of the age of rib fractures, with potential clinical and forensic applications.

**Key points:**

• Complex factors make it difficult to determine the age of a fracture.

• Our model based on radiomic features performed well in classifying fracture age.

• Associating the radiomic features with clinical features improved the model’s performance.

**Graphical Abstract:**

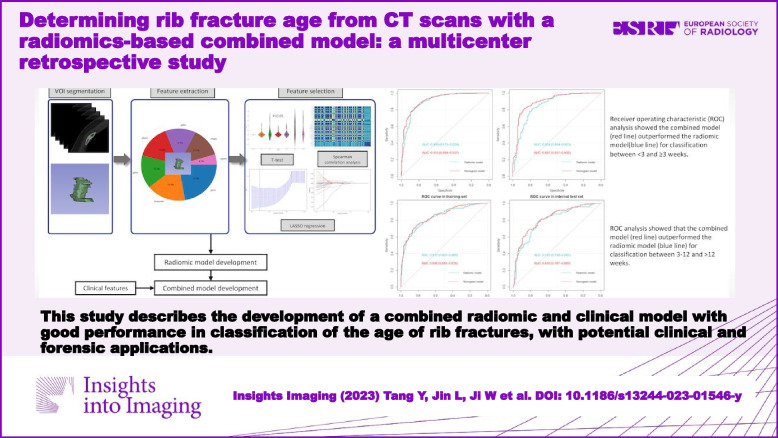

**Supplementary Information:**

The online version contains supplementary material available at 10.1186/s13244-023-01546-y.

## Introduction

Thoracic injuries are common among trauma patients [1]. Rib fractures are the most common form of thoracic trauma, serving as a marker of injury severity and a guide for legal decision-making [2, 3]. In the USA, 300,000 patients were diagnosed with rib fractures in 2004 [4], which increased to 350,000 in 2017 [5]. The risk of fracture increases significantly with age [6, 7]. Therefore, rib fracture cases are expected to increase with global aging [8]. The cause of rib fractures varies between age groups; they are usually caused by falls in older adults, traffic accidents in adults, and violent conflicts in adolescents [9, 10].

Accident compensation usually requires clarification of injury events leading to rib fractures [11]. In addition, the specific timing of rib fractures during accidents is important in legal cases. Providing the age of a rib fracture can clarify the association with a specific physical event and rule out irrelevant injuries, which is beneficial for settling compensation issues. Furthermore, the fracture healing state can be clinically assessed based on a patient’s radiological presentation and physical examination at different ages after the fracture [12]. However, this is influenced by multiple factors, including the patient’s age, nutritional status, sex, fracture location, and vascular injury, making it highly challenging to assess fracture union [13]. Combining imaging with fracture timing can improve surgeons’ confidence in assessing the healing state. Analyzing the fracture healing process can guide further treatment and care practices for patients with rib fractures [14].

Radiomics provides a new perspective for doctors by extracting quantitative features from medical images to reflect the heterogeneity of lesions that are unobservable by the naked eye. Its primary benefit lies in the quantitative and more objective interpretation of medical images, potentially overcoming the limitations of visual image assessment [15, 16]. To the best of our knowledge, no study has investigated whether a model derived from computed tomography (CT) imaging and clinical information can aid the diagnosis of fracture age. We aimed to evaluate the performance of a model built using radiomic and clinical data to diagnose fracture age.

## Methods

### Ethics consideration

This retrospective study was approved by the ethics committee of our hospital (No. 20220069), which waived the requirement for informed consent.

### Study population of the internal dataset

We searched the Picture Archiving and Communication System of our hospital using the keyword phrase “chest trauma evaluation” to identify patients who visited our hospital for chest trauma evaluation between March 2016 and September 2022. The inclusion criteria comprised the following: (1) patient age > 18 years; (2) patients who experienced a traumatic event (including traffic accidents, falls, and fights involving blunt chest trauma); (3) imaging reports confirming at least one rib fracture; (4) slice thickness after image reconstruction < 1.5 mm; and (5) case reports documenting the precise time of fractures. The exclusion criteria comprised the following: (1) malignant and benign bone tumors involving the ribs; (2) metastatic diseases involving the ribs; (3) significant artifacts in CT images; and (4) surgical internal fixation of rib fractures. We obtained demographic features, including age and sex, from the patient hospital records. The scan parameters are described in the Supplementary Material. We identified 71 patients with rib fractures imaged < 3 weeks after trauma and matched them by age to ensure that the number of fractures at 3–12 weeks and > 12 weeks was approximately equal to that at < 3 weeks. These three frames for depicting fracture ages were sourced from previously published literature [17, 18]. For each patient, 12 bilateral ribs were included in the dataset.

**Fig. 1:**
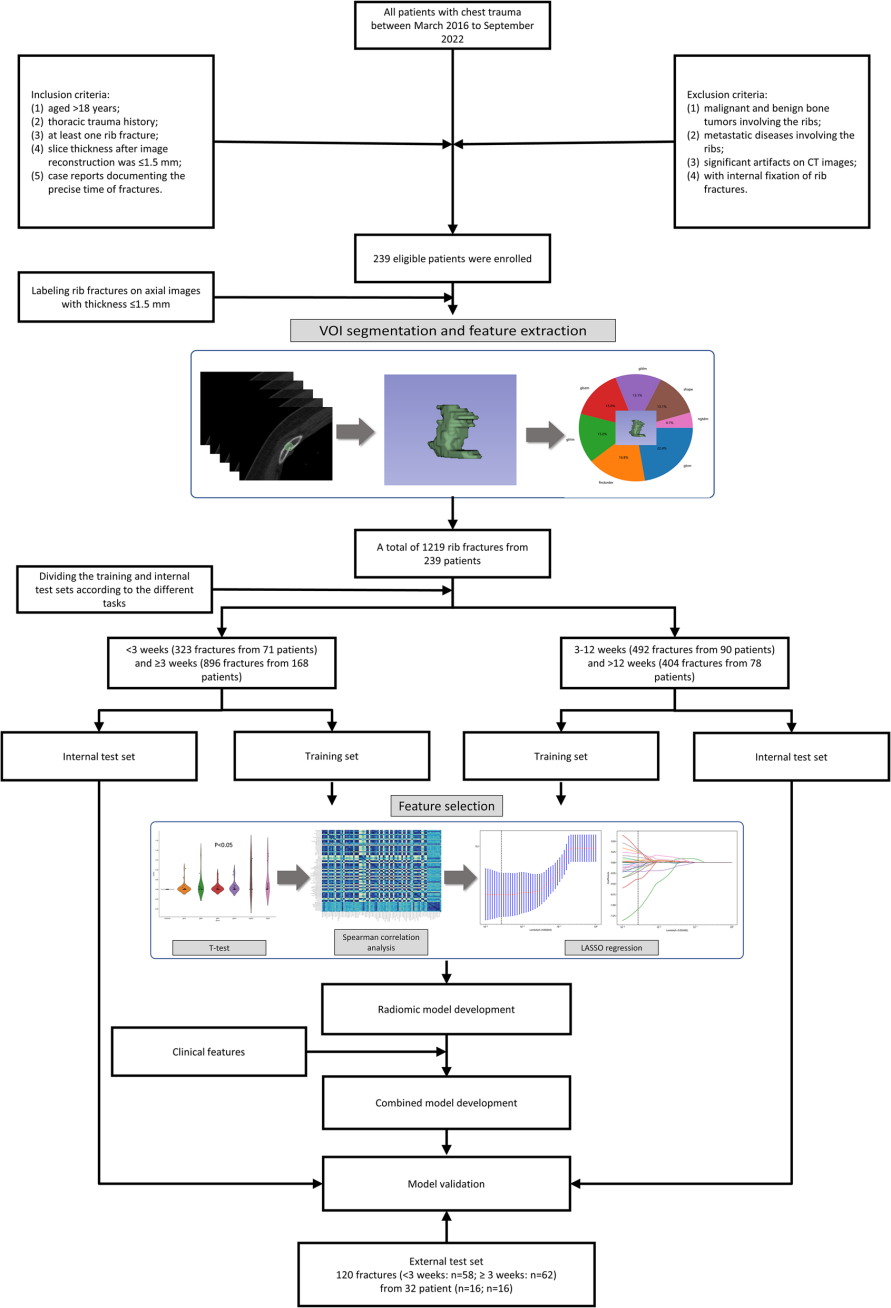
Flow chart of the recruitment pathway for the datasets used in this study

Finally, as shown in Fig. 1, based on our inclusion and exclusion criteria, 1219 rib fractures (< 3 weeks: *n* = 323; 3–12 weeks: *n* = 492; > 12 weeks: *n* = 404) from 239 patients (< 3 weeks: *n* = 71; 3–12 weeks: *n* = 90; > 12 weeks: *n* = 78) were included in our internal dataset. For classifying fracture age between < 3 and ≥ 3 weeks, 1219 fractures were randomly assigned to the training (*n* = 853) and internal test sets (*n* = 366). For classifying fracture age between 3–12 and > 12 weeks, 896 fractures were randomly assigned to the training (*n* = 627) and internal test sets (*n* = 269).

### Study population of the external dataset

To build a validation external dataset, we included 120 rib fractures from 32 patients (< 3 weeks: *n* = 16; ≥ 3 weeks: *n* = 16) who underwent chest trauma evaluation between October 2019 and August 2023, in another hospital in Shanghai according to the same inclusion and exclusion criteria to construct. This external dataset was used to confirm the efficacy of our model and assess the performance of radiologists in classifying rib fracture age based on CT images using the model. The scan parameters used for CT examination are provided in the Supplementary Material. Detailed clinical features are listed in Table S3.

### Extraction of radiomic and clinical features

Referring to the diagnostic CT reports, a radiologist with 5 years of experience in chest diagnostics manually delineated the volume of interest (VOI) at the fractured ends of the rib using the 3D Slicer software (https://www.slicer.org) (Figure S1). Subsequently, another radiologist with 20 years of experience in chest diagnostics confirmed the VOIs. Next, we used the PyRadiomics package in Python to extract radiomic features from the VOIs [19]. For each VOI, we extracted 107 radiomic features. The detailed outlining process and radiomic features are shown in the Supplementary Material.

The clinical features included demographic and CT image features. The clinical features were as follows: (1) pleural inflammation, (2) 1–4 rib fractures, (3) lateral fractures, (4) bone fragments, (5) multiple fractures of the same rib, (6) cartilage junction fractures, (7) periosteal callus information, (8) intramedullary callus information, and (9) sex.

### Selection of radiomic and clinical features

We hypothesized that meaningful features might differ when classifying fracture age between < 3 weeks, ≥ 3 weeks, 3–12 weeks, and > 12 weeks. Therefore, we selected the features separately according to these time frames.

All radiomic features were normalized using *z*-score normalization before feature selection. To minimize the impact of dimensionality, the selection of features was conducted in three steps using the training set. First, we calculated the *p* value for each feature using the *t*-test and selected features with *p* values < 0.05 for further consideration. Spearman’s correlation coefficients were calculated for the selected features to avoid the underlying severe linear dependence. When the value was < 0.9, we determined this to mean that there was no correlation between the selected features. Lastly, we used the least absolute shrinkage and selection operator (LASSO) analysis to determine the most useful radiomic features. Clinical features were selected using univariate and multivariate logistic regression analyses.

### Development of radiomic and combined models

We used two feature sets instead of one for the precise classification of the fracture age because of the difference in features for fracture age between < 3 weeks, ≥ 3 weeks, 3–12 weeks, and > 12 weeks. Regarding both levels of classification, we developed radiomic and combined models.

The base classifier acts as a feature encoder and has a significant impact on classification [20]. We compared the performance of five common machine learning (ML) algorithms, support vector machine (SVM), K-nearest neighbor, random forest, decision tree, and eXtreme Gradient Boosting to determine the most suitable classifier for fracture age assessment. Hyperparameters were optimized using grid search and tenfold cross-validation. Only the hyperparameters listed in Table S4 were adjusted, while all other hyperparameters were maintained at the default values specified by models. The filtered radiomic features were incorporated into the classifier and trained within the training set to obtain the radiomic model.

Using one of the two levels of binary classification (< 3 weeks or 3–12 weeks) as the reference standard, coded as 0 in the training of the classifier, we obtained the predicted probability of each fracture in the remaining two groups as the radiomic signature. Lastly, we developed the combined model based on the radiomic signature and clinical features using multivariate logistic regression.

### Nomogram building

In order to simplify the combined model into an easy-to-understand tool, a nomogram was utilized for constructing a simplified graphical display. The sum of nomogram points was calculated based on clinical features and radiomic signature. For the convenience of calculations, instantly deployable online calculators were developed.

### Statistical analysis

Statistical analysis was performed using SPSS 26.0 (IBM) and R Studio (ver. 4.3.1). Clinical features were measured using chi-squared or Fisher’s exact-probability testing as all were categorical variables. The clinical features with *p* values of < 0.05 in both univariate and multivariate logistic regression analyses were included in the combined model. Differences in radiomic features were assessed using the *t*-test or the Mann–Whitney *U* test as they were continuous variables. The statistical significance level was set at *p* values of < 0.05.

## Results

### Selection of clinical features

The clinical features were compared (Table S1 and S2), and we observed no significant differences between the clinical features of the training and internal test sets (*p* = 0.142–0.988). Results of univariate and multivariate logistic regression analyses for clinical features associated with the classification of fracture age are presented in Table 1. For classifying fracture age between < 3 and ≥ 3 weeks, six independent clinical features were selected, including sex, fractures of ribs 1–4, pleural inflammation, lateral fractures, intramedullary callus formation, and periosteal callus formation. For classifying fracture age between 3–12 and > 12 weeks, three independent clinical features were selected, including pleural inflammation, intramedullary callus formation, and multiple fractures of the same rib.

**Table 1:** Results of univariate and multivariate logistic regression analyses for classification of fracture age

	**Standard error**	**Odds ratio**	***p* value**
**Classification of fracture age (< 3 weeks vs ≥ 3 weeks)**			
**Results of univariate analysis**			
Age	0.007	1.012	0.117
Sex	0.170	0.667	0.017
Ribs 1–4 fractures	0.077	2.630	< 0.001
Pleural inflammation	0.209	0.257	< 0.001
Lateral fractures	0.198	2.267	< 0.001
Bone fragments	0.372	0.512	0.072
Multiple fractures of the same rib	0.160	1.582	0.004
Cartilage junction fractures	0.077	2.630	< 0.001
Periosteal callus information	0.077	2.630	< 0.001
Intramedullary callus information	0.180	6.706	< 0.001
**Positive results of multivariate logistic regression analysis**			
Sex	0.234	2.189	0.001
Ribs 1–4 fractures	0.199	2.060	< 0.001
Pleural inflammation	0.254	0.238	< 0.001
Lateral fractures	0.238	2.189	0.001
Periosteal callus formation	0.222	5.236	< 0.001
Intramedullary callus formation	3.565	3.565	< 0.001
**Classification of fracture age (3–12 weeks vs > 12 weeks)**			
**Results of univariate analysis**			
Age	0.009	1.010	0.274
Sex	0.168	0.595	0.020
Ribs 1–4 fractures	0.165	1.401	0.041
Pleural inflammation	0.399	0.246	< 0.001
Lateral fractures	0.172	1.273	0.161
Bone fragments	0.523	0.402	0.082
Multiple fractures of the same rib	0.165	0.492	< 0.001
Cartilage junction fractures	0.250	1.025	0.920
Periosteal callus formation	0.167	0.592	0.002
Intramedullary callus information	0.270	0.258	< 0.001
**Positive results of multivariate logistic regression analysis**			
Pleural inflammation	0.419	0.244	0.001
Multiple fractures of the same rib	0.175	0.528	< 0.001
Intramedullary callus formation	0.305	0.334	< 0.001

### Development and validation of the radiomic model

Using the < 3 weeks as the reference standard, 17 radiomic features were obtained using the *t*-test, Spearman’s correlation analysis, and LASSO regression analysis, including 3 shape, 4 intensity, and 10 texture features (Figure S2).

Using the 3–12 weeks as the reference standard, 10 radiomic features were obtained using the *t*-test, Spearman’s correlation analysis, and LASSO regression analysis, including 3 shape, 2 intensity, and 5 texture features (Figure S3).

SVM performed the best on fracture age assessment (Table S5); therefore, we chose it as the base classifier for the radiomic model and obtained the radiomic signature. Regarding the classification of the fracture age between < 3 weeks and ≥ 3 weeks, using < 3 weeks as the reference standard, the performance of the radiomic model showed an AUC of 0.897 (95% confidence interval [CI]: 0.875–0.925) and 0.854 (95% CI: 0.804–0.903) for the training and internal test sets, respectively. Regarding the classification of the fracture age between 3–12 weeks and > 12 weeks, using 3–12 weeks as the reference standard, the performance of the radiomic model showed an AUC of 0.837 (95% CI: 0.805–0.868) and 0.793 (95% CI: 0.738–0.847) for the training and internal test sets, respectively.

### Development and visualization of the combined model

**Fig. 2:**
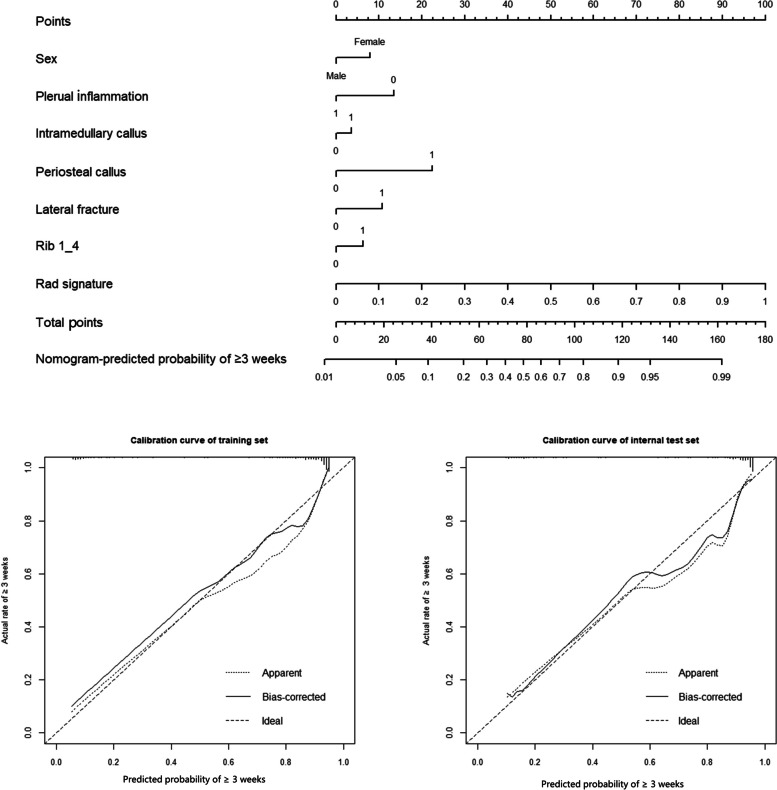
The combined model for classification between < 3 weeks and ≥ 3 weeks. The calibration curves of this model in the training and internal test sets were obtained by resampling 1000 times. The dotted line indicates the ideal ability, and the solid line represents the real ability of the model. The combined performed better when the solid line was closer to the dotted line

For classifying fracture age between < 3 and ≥ 3 weeks, using the selected clinical features and radiomic signature, a combined model was developed and visualized in the form of a nomogram (Fig. 2). The online version of the nomogram could be used at https://myr234r.shinyapps.io/DynNomapp3/, and its user interface is illustrated in Fig. 4a. The AUC values of the combined model were 0.914 (95% CI = 0.892–0.935) and 0.889 (95% CI = 0.846–0.932) for the training and internal test sets, respectively. The calibration curves revealed good predictive accuracy between the actual probability and predicted probability (Fig. 2).

**Fig. 3:**
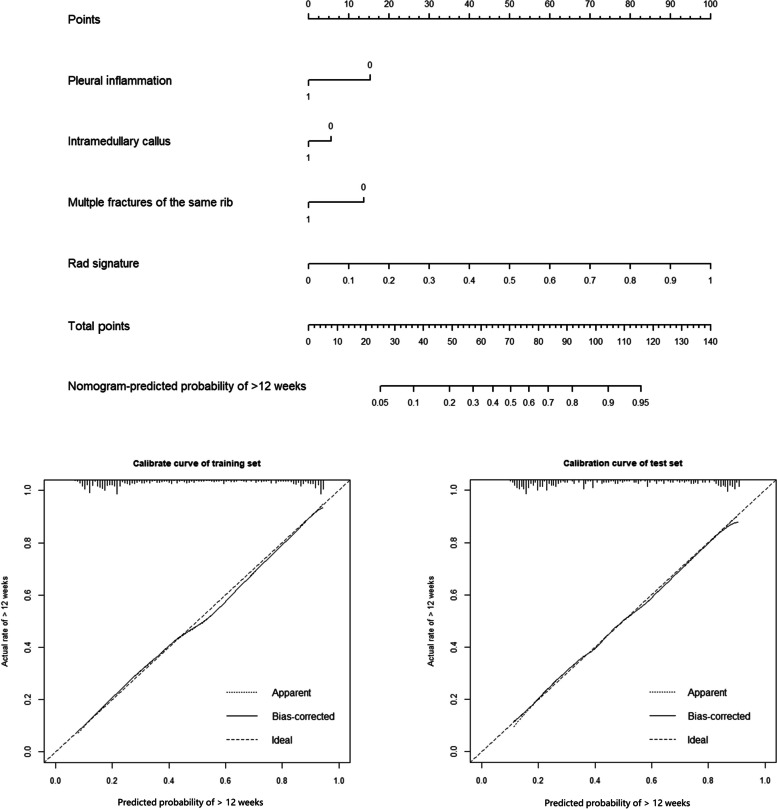
The combined model for classification between 3–12 weeks and > 12 weeks. The calibration curves of this model in the training and internal test sets were obtained by resampling 1000 times. The dotted line indicates the ideal ability, and the solid line represents the real ability of the model. The combined performed better when the solid line was closer to the dotted line

**Fig. 4:**
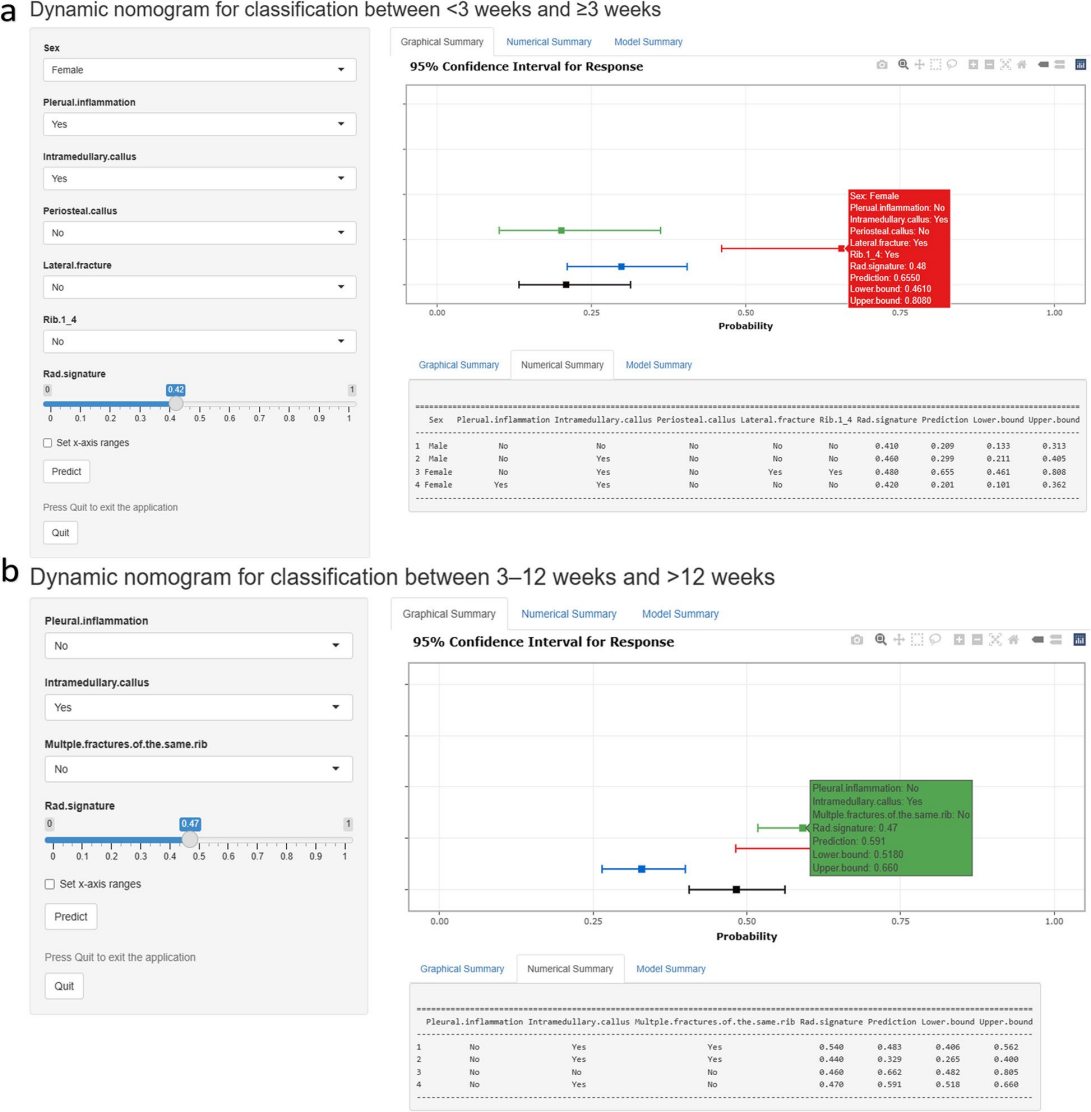
Online dynamic nomograms for classifying rib fracture age between < 3 and ≥ 3 weeks (**a**) and between 3–12 weeks and > 12 weeks (**b**). The figure displays the probabilities (with a 95% confidence interval) determined by the combined model for rib fracture ages of ≥ 3 weeks and > 12 weeks. The specific predicted values are provided in the “Numerical Summary” tab

For classifying fracture age between 3–12 and > 12 weeks, using the selected clinical features and radiomic signature, a combined model was developed and visualized in the form of a nomogram (Fig. 3). An online version of the nomogram could be used at https://myr234r.shinyapps.io/DynNomapp12/ and its user interface is illustrated in Fig. 4b. The AUC values of the combined model were 0.848 (95% CI: 0.818–0.878) and 0.818 (95% CI: 0.767–0.869) in the training and internal test sets, respectively. The calibration curves of the nomogram showed acceptable agreement between prediction and actual observation (Figs. 2 and 3).

### Performance comparison between the radiomic and combined models

The DeLong test showed that the combined model performed better than the radiomic model for classification between < 3 weeks, ≥ 3 weeks, 3–12 weeks, and > 12 weeks (Table 2).

**Table 2:** Performance comparison of the radiomic and combined models

**Model**		**AUC**	***p* value**	**SENS**	**SPEC**
** < 3 weeks vs ≥ 3 weeks**					
Radiomic model	T	0.900 (0.875–0.925)	Reference	0.857 (0.763–0.908)	0.855 (0.763–0.919)
	I-T	0.854 (0.804–0.903)		0.812 (0.74–0.924)	0.798 (0.663–0.876)
Combined model	T	0.915 (0.894–0.937)	0.009	0.851 (0.759–0.923)	0.838 (0.744–0.923)
	I-T	0.897 (0.857–0.938)	< 0.001	0.820 (0.729–0.903)	0.888 (0.775–0.995)
**3–12 weeks vs > 12 weeks**					
Radiomic model	T	0.837 (0.805–0.868)	Reference	0.729 (0.593–0.825)	0.855 (0.763–0.919)
	I-T	0.793 (0.738–0.847)		0.816 (0.740–0.931)	0.798 (0.663–0.876)
Combined model	T	0.848 (0.818–0.878)	0.12	0.732 (0.650–0.800)	0.836 (0.775–0.899)
	I-T	0.818 (0.767–0.869)	0.003	0.718 (0.597– 0.879)	0.821 (0.628–0.910)

*AUC* area under the receiver operating characteristic curve, *SENS* sensitivity, *SPEC* specificity, *T* training set, *I-T* internal test set

**Fig. 5:**
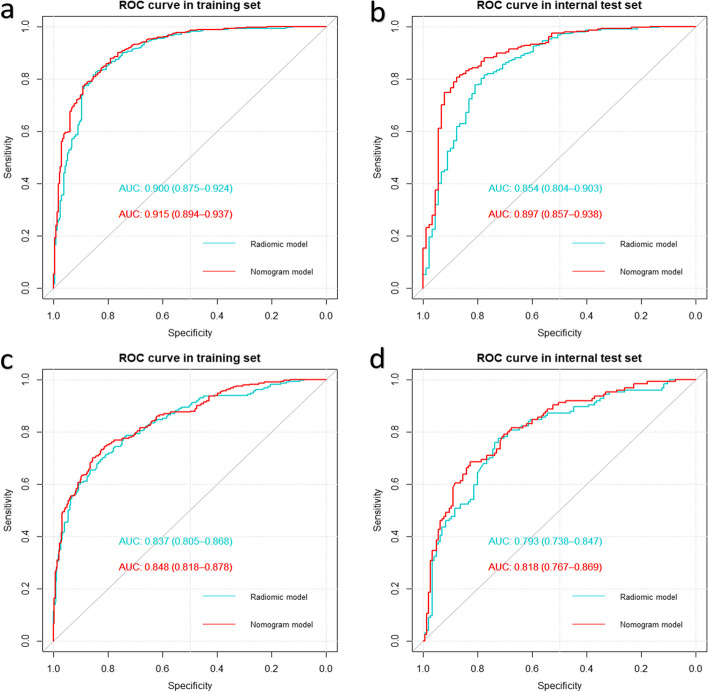
Receiver operating characteristic (ROC) analysis revealed that for the classification between < 3 and ≥ 3 weeks (**a**, **b**), as well as between 3–12 and > 12 weeks (**c**, **d**), the performance of the combined model (red line) surpassed that of the radiomics model (blue line) in both the training and internal test sets

For classifying fracture age between < 3 weeks and ≥ 3 weeks, the receiver operating characteristic** (**ROC) curves of the radiomic and combined models are shown in Fig. 5. In the training set, the combined model (AUC = 0.915, 95% CI = 0.893–0.936) outperformed the radiomic model (AUC = 0.900, 95% CI = 0.875–0.925; *p* = 0.009). Similarly, in the internal test set, the combined model (AUC = 0.889, 95% CI = 0.846–0.932) outperformed the radiomic model (AUC = 0.854, 95% CI = 0.804–0.903; *p* < 0.001).

For classifying the fracture age between 3–12 weeks and > 12 weeks, the ROC curves of the radiomic and combined models are shown in Fig. 5. In the training set, the AUC of the combined model (0.848, 95% CI = 0.818–0.878) was slightly higher than that of the radiomic model (0.837, 95% CI = 0.805–0.868; *p* = 0.12). However, in the internal test set, the combined model (AUC = 0.818, 95% CI = 0.767–0.869) performed significantly better than the radiomic model (AUC = 0.793, 95% CI = 0.738–0.847; *p* = 0.003).

### Performance in the external test set

In the external test set, we reached the same conclusion as that in the internal dataset (Table 3). We evaluated a radiologist’s (engaged in musculoskeletal imaging diagnosis for 15 years) performance in classifying rib fracture age aided by the combined model. For classifying fracture age between < 3 and ≥ 3 weeks, the AUC values for the radiomic model, combined model, and radiologist aided by the combined model were 0.811 (95% CI = 0.733–0.889), 0.881 (95% CI = 0.814–0.949), and 0.966 (95% CI = 0.935–0.996), respectively. This indicated that the combined model was better than the radiomic model, and the collaborative integration of the radiologist with the combined model further enhanced classification performance (Figure S4).

**Table 3:** Performance comparison of the radiomic model, combined model, and nomogram-assisted radiologist for classifying fracture age between < 3 and ≥ 3 weeks in the external test set

Model	AUC	*p* value	SENS	SPEC
Radiomic model	0.811 (0.733–0.889)	0.003	0.871 (0.548–0.968)	0.690 (0.535–0.966)
Combined model	0.881 (0.814–0.949)	Reference	0.887 (0.742–0.968)	0.862(0.724–0.966)
Radiologist aided by the combined model	0.966 (0. 935–0.996)	0.007	0.984 (0.887–1)	0.897 (0.793–0.966)

*AUC* area under the receiver operating characteristic curve, *SENS* sensitivity, *SPEC* specificity

## Discussion

The results from the current study showed that our combined model, based on clinical and radiomic features, could accurately classify the age of rib fractures. The good performance of the radiologist assisted by the model demonstrated the potential feasibility of the model in practical applications, and it is expected to provide value for clinical and forensic decision-making.

After a fracture, the fracture region undergoes a series of characteristic events over time called the inflammatory, repair, and remodeling phases, all of which are influenced by many complex factors [21, 22]. Sex, 1–4 rib fractures, and lateral fractures impact fracture healing through estrogen levels, vascular injury, and respiration and body movements, respectively [14, 23–26]. Pleural inflammation and callus formation are considered important imaging features of the inflammatory and reparative phases of fracture healing, respectively [21, 27]. In late fracture healing, multiple fractures of the same rib end healing early by promoting bone resorption [28]. Therefore, the above clinical features were included in our combined model. Radiomic enables the quantification of heterogeneity of fracture regions, thereby compensating for the limitations of visual image assessment. The combination of all pertinent features (visually observable clinical features and visually difficult-to-identify radiomic features) in the combined model allows for a comprehensive assessment of fracture regions, providing radiologists with additional data-driven insights. Thus, aided by the combined model, radiologists achieve diagnoses by integrating subjective and objective interpretations of medical images, together with their individual clinical experience. This fusion of knowledge from both human expertise and data-driven analysis enhances the diagnostic decision-making process, ultimately improving diagnostic performance.

Radiomics are useful for detecting changes in bone microarchitecture due to injury and aging [29–32]. Several studies [33–35] utilized radiomic features from T2-weighted images to achieve early diagnosis of osteoarthritis. Lin et al. [36] developed a radiomic and clinical features-based model to predict the prognosis of osteoarthritis, with an AUC of 0.83 (95% CI = 0.70–0.96) in the validation set. Recent studies [37–45] also demonstrated the ability of radiomics in osteoporosis detection and fracture prediction. Apart from its applications in diagnosing bone disorders, radiomics research is starting to emerge in the field of forensic medicine. Giorgio et al. [46] demonstrated, based on a limited sample (*n* = 4), that CT-based radiomics could be associated with time of death. Subsequently, Klontzas et al. [47] applied CT-based radiomics to predict postmortem interval, yielding promising results in this exploratory study (AUC = 0.75, 95%CI = 0.584–0.916). A previous forensic study [48] indicated an association between CT-based quantitative parameters and the age of rib fractures. However, the limited sample size (*n* = 9) and the finite number of extracted features (*n* = 5) were insufficient for conducting ML analysis. The aforementioned radiomics research in forensic medicine predominantly focused on postmortem forensic investigations, whereas this study pertained to trauma assessment for legal proceedings. Our study was based on principles similar to those of the aforementioned studies, namely, that radiomics, which reflect changes in bone microstructure during the bone healing process, can be used for fracture age classification, and that the addition of clinical features can further enhance model performance.

A strength of this study is the development of a single radiomic signature that summarizes various radiomic features, providing a useful tool for clinical practice. In addition, the combined model was visualized as a nomogram, providing readers with more tangible interpretation of each factor’s impact on the classification. We have made our combined model public on an open-access website in the form of online calculator to simplify the calculations. However, some limitations exist in our study. First, this was a retrospective study. A prospective study is needed to further validate our model. Second, older participants were included in this study (median age, 57 years; interquartile range, 50–63 years) and the model’s applicability to younger patients is unknown. Younger patients should be included in future studies to improve the robustness of the model. Lastly, in the absence of data > 12 weeks from the external center, we did not perform external validation for the model in classifying fracture age between 3–12 and > 12 weeks. Future studies could be designed to overcome these limitations.

## Conclusions

Our study established a combined model for rib fracture age classification based on CT images. The combined model and the model-assisted radiologist achieved good performance in classifying rib fracture age. This model has the potential to influence clinical practice and forensic decision-making.

### Supplementary Information

**Additional file 1: Supplementary methods. Table S1.** Baseline characteristics of the internal dataset for classification between <3 and ≥3 weeks. **Table S2.** Baseline characteristics of the internal dataset for classification between 3‒12 weeks and >12 weeks. **Table S3. **Baseline characteristics of the external dataset. **Table S4. **Optimal hyperparameter configuration list for machine learning algorithms. **Table S5.** Performance comparison of different models. **Figure S1.** Delineation of the volume of interest (VOI) for the rib fracture region. **Figure S2.** LASSO regression analysis procedure for the selection of radiomic features. **Figure S3.** LASSO regression analysis procedure for the selection of radiomic features. **Figure S4.** Receiver operating characteristic (ROC) comparison among radiomic model, combined model, and nomogram-assisted radiologist using the external test set.

## Data Availability

All the data will be shared upon reasonable request by the corresponding author.

## Abbreviations

AUC: Area under the curve
CI: Confidence interval
LASSO: Least absolute shrinkage and selection operator
OR: Odds ratio
ROC: Receiver operating characteristic
SVM: Support vector machine
VOI: Volume of interest

## References

[1] Head W, Kumar N, Thomas C, Leon S, Dieffenbaugher S, Eriksson E (2021). Are rib fractures stable? An analysis of progressive rib fracture offset in the acute trauma setting. J Trauma Acute Care Surg.

[2] O'Donovan S, van den Heuvel C, Baldock M, Humphries MA, Byard RW (2022). Fatal blunt chest trauma: an evaluation of rib fracture patterns and age. Int J Legal Med.

[3] Yu L, Baumann BM, Raja AS (2020). Blunt traumatic aortic injury in the pan-scan era. Acad Emerg Med.

[4] Lafferty PM, Anavian J, Will RE, Cole PA (2011). Operative treatment of chest wall injuries: indications, technique, and outcomes. J Bone Joint Surg Am.

[5] Pieracci FM, Majercik S, Ali-Osman F (2017). Consensus statement: surgical stabilization of rib fractures rib fracture colloquium clinical practice guidelines. Injury.

[6] Ensrud KE (2013). Epidemiology of fracture risk with advancing age. J Gerontol A Biol Sci Med Sci.

[7] Bonicelli A, Kranioti EF, Xhemali B, Arnold E, Zioupos P (2022). Assessing bone maturity: compositional and mechanical properties of rib cortical bone at different ages. Bone.

[8] He Z, Zhang D, Xiao H (2019). The ideal methods for the management of rib fractures. J Thorac Dis.

[9] Coary R, Skerritt C, Carey A, Rudd S, Shipway D (2020). New horizons in rib fracture management in the older adult. Age Ageing.

[10] Chua MT, Pan DST, Lee MZ (2022). Epidemiology and outcomes of older trauma patients in Singapore: a multicentre study. Injury.

[11] Lau G, Gabbe BJ, Giummarra MJ (2021). Patterns and predictors of personal responsibility attributions after major trauma. Injury.

[12] Morshed S (2014). Current options for determining fracture union. Adv Med.

[13] Zura R, Xu ZJ, Della Rocca GJ, Mehta S, Steen RG (2017). When is a fracture not “fresh”? Aligning reimbursement with patient outcome after treatment with low-intensity pulsed ultrasound. J Orthop Trauma.

[14] Fisher JS, Kazam JJ, Fufa D, Bartolotta RJ (2019). Radiologic evaluation of fracture healing. Skeletal Radiol.

[15] Gillies RJ, Kinahan PE, Hricak H (2016). Radiomics: images are more than pictures, they are data. Radiology.

[16] Abbasian Ardakani A, Bureau NJ, Ciaccio EJ, Acharya UR (2022). Interpretation of radiomics features-a pictorial review. Comput Methods Programs Biomed.

[17] Wu X, Jiang Y (2015). Old fracture. Zhonghua Wai Ke Za Zhi.

[18] Arredondo-Gómez E (2007). Treatment of traumatic clavicular pseudoarthrosis with the Hunec Colchero nail. Acta Ortop Mex.

[19] van Griethuysen JJM, Fedorov A, Parmar C (2017). Computational radiomics system to decode the radiographic phenotype. Cancer Res.

[20] Deist TM, Dankers F, Valdes G (2018). Machine learning algorithms for outcome prediction in (chemo)radiotherapy: an empirical comparison of classifiers. Med Phys.

[21] Claes L, Recknagel S, Ignatius A (2012). Fracture healing under healthy and inflammatory conditions. Nat Rev Rheumatol.

[22] Palanisamy P, Alam M, Li S, Chow SKH, Zheng YP (2022). Low-intensity pulsed ultrasound stimulation for bone fractures healing: a review. J Ultrasound Med.

[23] Talbot BS, Gange CP, Chaturvedi A, Klionsky N, Hobbs SK, Chaturvedi A (2017). Traumatic rib injury: patterns, imaging pitfalls, complications, and treatment. Radiographics.

[24] Claes L (2011). Biomechanical principles and mechanobiologic aspects of flexible and locked plating. J Orthop Trauma.

[25] Rizzoli R, Reginster JY, Boonen S (2011). Adverse reactions and drug-drug interactions in the management of women with postmenopausal osteoporosis. Calcif Tissue Int.

[26] Fischer V, Haffner-Luntzer M (2022). Interaction between bone and immune cells: implications for postmenopausal osteoporosis. Semin Cell Dev Biol.

[27] Kolar P, Schmidt-Bleek K, Schell H (2010). The early fracture hematoma and its potential role in fracture healing. Tissue Eng Part B Rev.

[28] Loi F, Córdova LA, Pajarinen J, Lin TH, Yao Z, Goodman SB (2016). Inflammation, fracture and bone repair. Bone.

[29] Le Corroller T, Halgrin J, Pithioux M, Guenoun D, Chabrand P, Champsaur P (2012). Combination of texture analysis and bone mineral density improves the prediction of fracture load in human femurs. Osteoporos Int.

[30] Xuan A, Chen H, Chen T (2023). The application of machine learning in early diagnosis of osteoarthritis: a narrative review. Ther Adv Musculoskelet Dis.

[31] Fritz B, Yi PH, Kijowski R, Fritz J (2023). Radiomics and deep learning for disease detection in musculoskeletal radiology: an overview of novel MRI- and CT-based approaches. Invest Radiol.

[32] Levi R, Garoli F, Battaglia M (2023). CT-based radiomics can identify physiological modifications of bone structure related to subjects' age and sex. Radiol Med.

[33] Peuna A, Hekkala J, Haapea M (2018). Variable angle gray level co-occurrence matrix analysis of T(2) relaxation time maps reveals degenerative changes of cartilage in knee osteoarthritis: Oulu knee osteoarthritis study. J Magn Reson Imaging.

[34] Xie Y, Dan Y, Tao H (2021). Radiomics feature analysis of cartilage and subchondral bone in differentiating knees predisposed to posttraumatic osteoarthritis after anterior cruciate ligament reconstruction from healthy knees. Biomed Res Int.

[35] Hirvasniemi J, Klein S, Bierma-Zeinstra S, Vernooij MW, Schiphof D, Oei EHG (2021). A machine learning approach to distinguish between knees without and with osteoarthritis using MRI-based radiomic features from tibial bone. Eur Radiol.

[36] Lin T, Peng S, Lu S (2023). Prediction of knee pain improvement over two years for knee osteoarthritis using a dynamic nomogram based on MRI-derived radiomics: a proof-of-concept study. Osteoarthritis Cartilage.

[37] Farzi M, Pozo JM, McCloskey E (2022). Quantitating age-related BMD textural variation from DXA region-free-analysis: a study of hip fracture prediction in three cohorts. J Bone Miner Res.

[38] Hong N, Park H, Kim CO (2021). Bone radiomics score derived from DXA hip images enhances hip fracture prediction in older women. J Bone Miner Res.

[39] Rastegar S, Vaziri M, Qasempour Y (2020). Radiomics for classification of bone mineral loss: a machine learning study. Diagn Interv Imaging.

[40] Huang CB, Hu JS, Tan K, Zhang W, Xu TH, Yang L (2022). Application of machine learning model to predict osteoporosis based on abdominal computed tomography images of the psoas muscle: a retrospective study. BMC Geriatr.

[41] Dai H, Wang Y, Fu R (2023). Radiomics and stacking regression model for measuring bone mineral density using abdominal computed tomography. Acta Radiol.

[42] Zhang J, Liu J, Liang Z (2023). Differentiation of acute and chronic vertebral compression fractures using conventional CT based on deep transfer learning features and hand-crafted radiomics features. BMC Musculoskelet Disord.

[43] Biamonte E, Levi R, Carrone F (2022). Artificial intelligence-based radiomics on computed tomography of lumbar spine in subjects with fragility vertebral fractures. J Endocrinol Invest.

[44] Zhang H, Yuan G, Wang C (2023). Differentiation of benign versus malignant indistinguishable vertebral compression fractures by different machine learning with MRI-based radiomic features. Eur Radiol.

[45] Chen YC, Li YT, Kuo PC (2023). Automatic segmentation and radiomic texture analysis for osteoporosis screening using chest low-dose computed tomography. Eur Radiol.

[46] De-Giorgio F, Ciasca G, Fecondo G (2022). Post mortem computed tomography meets radiomics: a case series on fractal analysis of post mortem changes in the brain. Int J Legal Med.

[47] Klontzas ME, Leventis D, Spanakis K, Karantanas AH, Kranioti EF (2023) Post-mortem CT radiomics for the prediction of time since death. Eur Radiol 33(11):8387–8395. 10.1007/s00330-023-09746-2 37329460

[48] Viero A, Biehler-Gomez L, Messina C (2022). Utility of micro-CT for dating post-cranial fractures of known post-traumatic ages through 3D measurements of the trabecular inner morphology. Sci Rep.

