# Supplementary material for: Determining rib fracture age from CT scans with a radiomics-based combined model: a multicenter retrospective study
Source: Insights Imaging. 2023 Dec 10;14:214. doi: 10.1186/s13244-023-01546-y (PMC10710975; doi:10.1186/s13244-023-01546-y)
Supplement: Supplementary file 1 — Additional file 1: Supplementary methods. Table S1. Baseline characteristics of the internal dataset for classification between <3 and ≥3 weeks. Table S2. Baseline characteristics of the internal dataset for classification between 3‒12 weeks and >12 weeks. Table S3. Baseline characteristics of the external dataset. Table S4. Optimal hyperparameter configuration list for machine learning algorithms. Table S5. Performance comparison of different models. Figure S1. Delineation of the volume of interest (VOI) for the rib fracture region. Figure S2. LASSO regression analysis procedure for the selection of radiomic features. Figure S3. LASSO regression analysis procedure for the selection of radiomic features. Figure S4. Receiver operating characteristic (ROC) comparison among radiomic model, combined model, and nomogram-assisted radiologist using the external test set. [file 13244_2023_1546_MOESM1_ESM.docx]

**Determining rib fracture age from CT scans with a radiomics-based combined model: a multicenter retrospective study**

**ELECTRONIC SUPPLEMENTARY MATERIAL**

**Supplementary methods**

Image acquisition in the internal dataset

Briefly, images were acquired using three different CT scanners: a 16-cm-wide coverage detector CT scanner (Revolution CT, GE Healthcare, WI, USA), dual-source CT scanner (Somatom Definition Flash, Siemens Healthcare, Germany), and dual-source CT scanner (Somatom Definition Drive, Siemens Healthcare). The following parameters were used: 100‒120 kVp depending on the patient’s size; 100–200 mAs; pitch, 0.75‒1.5; and collimation, 1‒1.25 mm, respectively. All imaging data were reconstructed using a bone or medium-sharp reconstruction algorithm to a 512 × 512 matrix with a thickness of 1‒1.5 mm.

Image acquisition in the external dataset

Briefly, images were acquired using a second dual-source CT scanner (Somatom Definition Flash, Siemens Healthcare). The acquisition parameters were as follows: 100‒120 kVp depending on the patient’s size; 100 effective mA with dose modulation; pitch, 0.75‒1.5. All imaging scan data were reconstructed using B60f or B50f reconstruction kernels to a 512 × 512 matrix, using a slice thickness of 1 mm.

Image segmentation and extraction of radiomic features

To ensure that all images were analyzed in the same window level, the window level and width of all CT images were set to 500 and 1500, respectively, before segmenting the fracture region and recording clinical features. Regarding the diagnostic CT report, combined with the display of the rib fracture area in the coronal, sagittal, and cross-sectional CT images, the volume of interest (VOI) for rib fractures on axial images was manually delineated for each study participant (Figure S1). In cases where there is no presence of callus within the fracture region, the VOI should be defined to include the rib fracture ends and the fracture gap as comprehensively as possible. In instances where callus formation is evident within the fracture area, the VOI should encompass not only the rib fracture ends but also extend to cover the region where callus has formed. Additionally, small fragments within the fracture area are regarded as integral constituents of the rib fracture region and should not be excluded during the delineation of the VOI. Next, we used PyRadiomics package in Python to extract radiomic features from the CT images and VOIs. For each VOI, we extracted 107 radiomic features. These radiomic features were divided into three groups: (I) geometry, (II) intensity, and (III) texture. The geometric and intensity features described the three-dimensional shape characteristics of the VOI and the first-order statistical distribution of voxel intensities within the VOI, respectively. Moreover, the texture features described the patterns or the second and high-order spatial distributions of the intensities. We extracted the texture features using different methods, including the gray-level co-occurrence matrix (GLCM), gray-level run length matrix (GLRLM), gray-level size zone matrix, and neighborhood gray-tone difference matrix (NGTDM) methods. The final extracted features were as follows: (1) first-order statistics (n = 18), (2) shape (n = 14), (3) GLCM (n = 24), (4) GLDM (n = 14), (5) GLSM (n = 16), (6) NGTDM (n = 5), and (7) GLRLM (n = 16).

**Table S1.** Baseline characteristics of the internal dataset for classification between <3 and ≥3 weeks.

| Characteristics | Training set (n=853) | Internal test set (n=366) | P value |
| --- | --- | --- | --- |
| Fracture time |  |  | 0.204 |
| <3 weeks | 235 (27.5%) | 88 (24%) |  |
| ≥3 weeks | 618 (72.5%) | 278 (76%) |  |
| Age, years | 57 (50,63) | 56 (50,63) | 0.724 |
| Sex |  |  |  |
| Male | 571 (66.9%) | 235 (64.2%) | 0.356 |
| Female | 282 (33.1%) | 131 (35.8%) |  |
| 1‒4 rib fractures |  |  | 0.312 |
| - | 550 (64.5%) | 247 (67.5%) |  |
| + | 303 (35.5%) | 119 (32.5%) |  |
| Multiple fractures of the same rib |  |  | 0.988 |
| - | 503 (59%) | 216 (59%) |  |
| + | 350 (41%) | 366 (41%) |  |
| Pleural inflammation |  |  |  |
| - | 741 (86.9%) | 325 (88.8%) | 0.352 |
| + | 112 (13.1%) | 41 (11.2%) |  |
| Lateral fractures |  |  | 0.202 |
| - | 627 (72.4%) | 256 (69.6%) |  |
| + | 226 (26.5%) | 110 (30.1%) |  |
| Bone fragments |  |  | 0.406 |
| - | 833 (96.4%) | 349 (95.4%) |  |
| + | 31 (3.6%) | 17 (4.6) |  |
| Cartilage junction fractures |  |  | 0.253 |
| - | 732 (85.8%) | 323 (88.3%) |  |
| + | 121 (14.2%) | 43 (11.7%) |  |
| Periosteal callus formation |  |  | 0.925 |
| - | 429 (50.3%) | 183 (50%) |  |
| + | 424 (49.7%) | 183 (50%) |  |
| Slice thickness | 1.063 ± 0.113 | 1.077 ± 0.123 | 0.5 |
| Intramedullary callus formation |  |  | 0.239 |
| - | 188 (22%) | 92 (25.1%） |  |
| + | 665 (78%) | 274 (74.9%) |  |

**Table S2.** Baseline characteristics of the internal dataset for classification between 3‒12 weeks and >12 weeks.

| Characteristics | Training set (n=627) | Internal test set (n=269) | P value |
| --- | --- | --- | --- |
| Fracture time |  |  | 0.691 |
| 3‒12 weeks | 347 (55.3%) | 145 (53.9%) |  |
| >12 weeks | 280 (44.7%) | 124 (46.1%) |  |
| Age, years | 57 (52,63) | 56 (51,63) | 0.653 |
| Sex |  |  | 0.188 |
| Male | 402 (64.1%) | 160 (59.5%) |  |
| Female | 225 (35.9%) | 109 (40.5%) |  |
| 1‒4 rib fractures |  |  | 0.644 |
| - | 386 (61.6%) | 170 (63.2%) |  |
| + | 241 (38.4%) | 99 (36.8%) |  |
| Multiple fractures of the same rib |  |  | 0.770 |
| - | 350 (55.8%) | 153 (56.9%) |  |
| + | 277 (44.2%) | 116 (43.1%) |  |
| Pleural inflammation |  |  |  |
| - | 582 (92.8%) | 246 (91.4%) | 0.447 |
| + | 45 (7.2%) | 23 (8.6%) |  |
| Lateral fractures |  |  | 0.967 |
| - | 428 (68.3%) | 184 (68.4%) |  |
| + | 199 (31.7%) | 85 (31.6%) |  |
| Bone fragments |  |  | 0.5 |
| - | 607 (96.8%) | 258 (95.9%) |  |
| + | 20 (3.2%) | 11 (4.1%) |  |
| Cartilage junction fractures |  |  | 0.710 |
| - | 554 (88.4%) | 240 (89.2%) |  |
| + | 73 (11.6%) | 29 (10.8%) |  |
| Periosteal callus formation |  |  | 0.142 |
| - | 233 (37.2%) | 114 (42.4%) |  |
| + | 394 (62.8%) | 155 (57.6%) |  |
| Slice thickness | 1.067 ± 0.118 | 1.064 ± 0.116 | 0.511 |
| Intramedullary callus formation |  |  | 0.292 |
| - | 77 (12.3%) | 40 (14.9%) |  |
| + | 550 (87.7%) | 229 (85.1%) |  |

**Table S3.** Baseline characteristics of the external dataset.

| Characteristics | External test set (n=120) |
| --- | --- |
| Fracture time |  |
| <3 weeks | 58 (48.3%) |
| ≥3 weeks | 62 (51.7%) |
| Age, years | 67 (55,77.25) |
| Sex |  |
| Male | 52 (43.3%) |
| Female | 68 (56.7%) |
| Slice thickness | 1 (100%) |
| 1‒4 rib fractures |  |
| - | 77(64.2%) |
| + | 43 (35.8%) |
| Multiple fractures of the same rib |  |
| - | 70 (58.3%) |
| + | 50 (41.7%) |
| Bone fragments |  |
| - | 114 (95.0%) |
| + | 6 (5.0%) |
| Periosteal callus formation |  |
| - | 85 (70.8%) |
| + | 35 (29.2%) |
| Pleural inflammation |  |
| - | 95 (79.2%) |
| + | 25 (20.8%) |
| Lateral fractures |  |
| - | 93 (77.5%) |
| + | 27 (22.5%) |
| Cartilage junction fractures |  |
| - | 103(85.8%) |
| + | 17 (14.2%) |
| Intramedullary callus formation |  |
| - | 78 (65.0%) |
| + | 42 (35.0%) |

**Table S4.** Optimal hyperparameter configuration list for machine learning algorithms.

| Model name | Hyperparameter |
| --- | --- |
| SVM | probability=True |
| KNN | algorithm=” kd_tree” |
| Random forest | n_estimators=10  max_depth=None  min_samples_split=2 |
| Decision tree | max_depth=None  min_samples_split=2 |
| XG Boost | n_estimators=10  objectives=” binary: logistic”  use_label_encoder=False  eval_metric=” error” |

SVM, support vector machine; KNN, K-nearest neighbor; XG boost, eXtreme Gradient Boosting.

**Table S5.** Performance comparison of different models.

|  |  | AUC | SENS | SPEC |
| --- | --- | --- | --- | --- |
| **<3 weeks vs ≥ 3 weeks** | | | | |
| SVM | T | 0.897 (0.875-0.925) | 0.857 (0.763-0.908) | 0.855 (0.763-0.919) |
|  | I-T | 0.854 (0.804-0.903) | 0.816 (0.74-0.931) | 0.798 (0.663-0.876) |
| KNN | T | 0.93 (0.915-0.946) | 0.838 (0.809-0.866) | 0.868 (0.825-0.906) |
|  | I-T | 0.802 (0.746-0.858) | 0.794 (0.74-0.895) | 0.73 (0.607-0.82) |
| Random forest | T | 1 (0.999-1) | 0.99 (0.981-0.998) | 1 (0.992-1) |
|  | I-T | 0.843 (0.796-0.89) | 0.726 (0.672-0.823) | 0.876 (0.764-0.944) |
| Decision tree | T | 1 (1-1) | 1 (1-1) | 1 (1-1) |
|  | I-T | 0.844 (0.799-0.89) | 0.729 (0.672-0.83) | 0.854 (0.742-0.933) |
| XG Boost | T | 0.999 (0.998-1) | 0.989 (0.966-0.997) | 0.992 (0.974-1) |
|  | I-T | 0.848 (0.802-0.894) | 0.78 (0.708-0.838) | 0.832 (0.73-0.899) |
| **3**‒**12 weeks vs. >12 weeks** | | | | |
| SVM | T | 0.837 (0.805-0.868) | 0.729 (0.593-0.825) | 0.813 (0.718-0.922) |
|  | I-T | 0.793 (0.738-0.847) | 0.79 (0.677-0.879) | 0.724 (0.621-0.821) |
| KNN | T | 0.88 (0.855-0.905) | 0.746 (0.696-0.943) | 0.842 (0.62-0.879) |
|  | I-T | 0.724 (0.664-0.783) | 0.645 (0.411-0.887) | 0.703 (0.435-0.903) |
| Random forest | T | 0.999 (0.998-1) | 0.996 (0.982-1) | 0.983 (0.965-0.994) |
|  | I-T | 0.701 (0.64-0.762) | 0.79 (0.46-0.984) | 0.524 (0.283-0.841) |
| Decision tree | T | 1 (1-1) | 1 (1-1) | 1 (1-1) |
|  | I-T | 0.75 (0.693-0.808) | 0.629 (0.5-0.871) | 0.779 (0.497-0.876) |
| XG Boost | T | 0.988 (0.983-0.994) | 0.936 (0.896-0.971) | 0.963 (0.919-0.989) |
|  | I-T | 0.739 (0.68-0.798) | 0.758 (0.613-0.871) | 0.676 (0.524-0.8) |

95% confidence intervals are included in brackets.

AUC, area under the receiver operating characteristic curve; SENS, sensitivity; SPEC, specificity; T, training set; I–T, internal test set; SVM, support vector machine; KNN, K-nearest neighbor; XG boost, eXtreme Gradient Boosting.


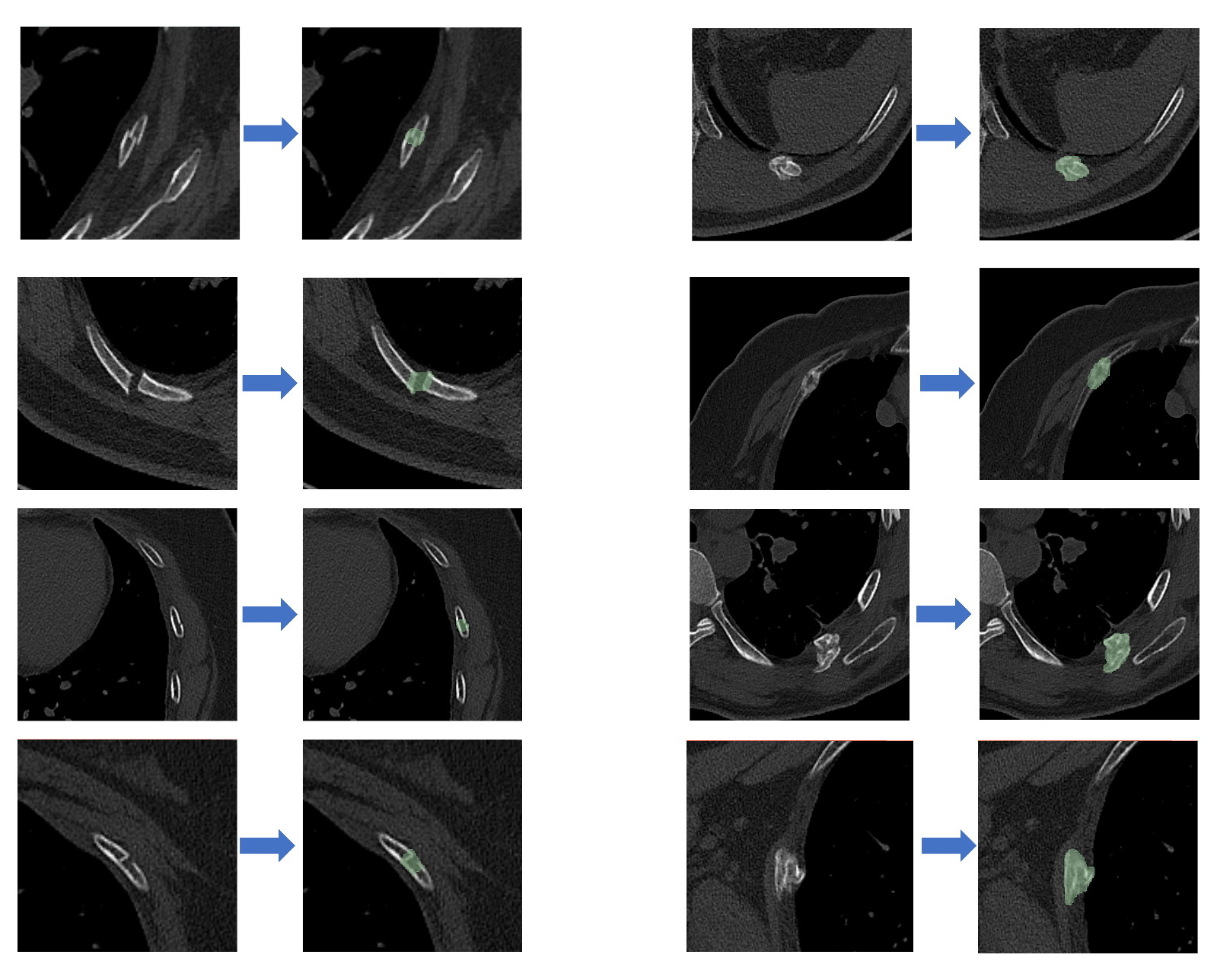


**Figure S1.** Delineation of the volume of interest (VOI) for the rib fracture region.

**
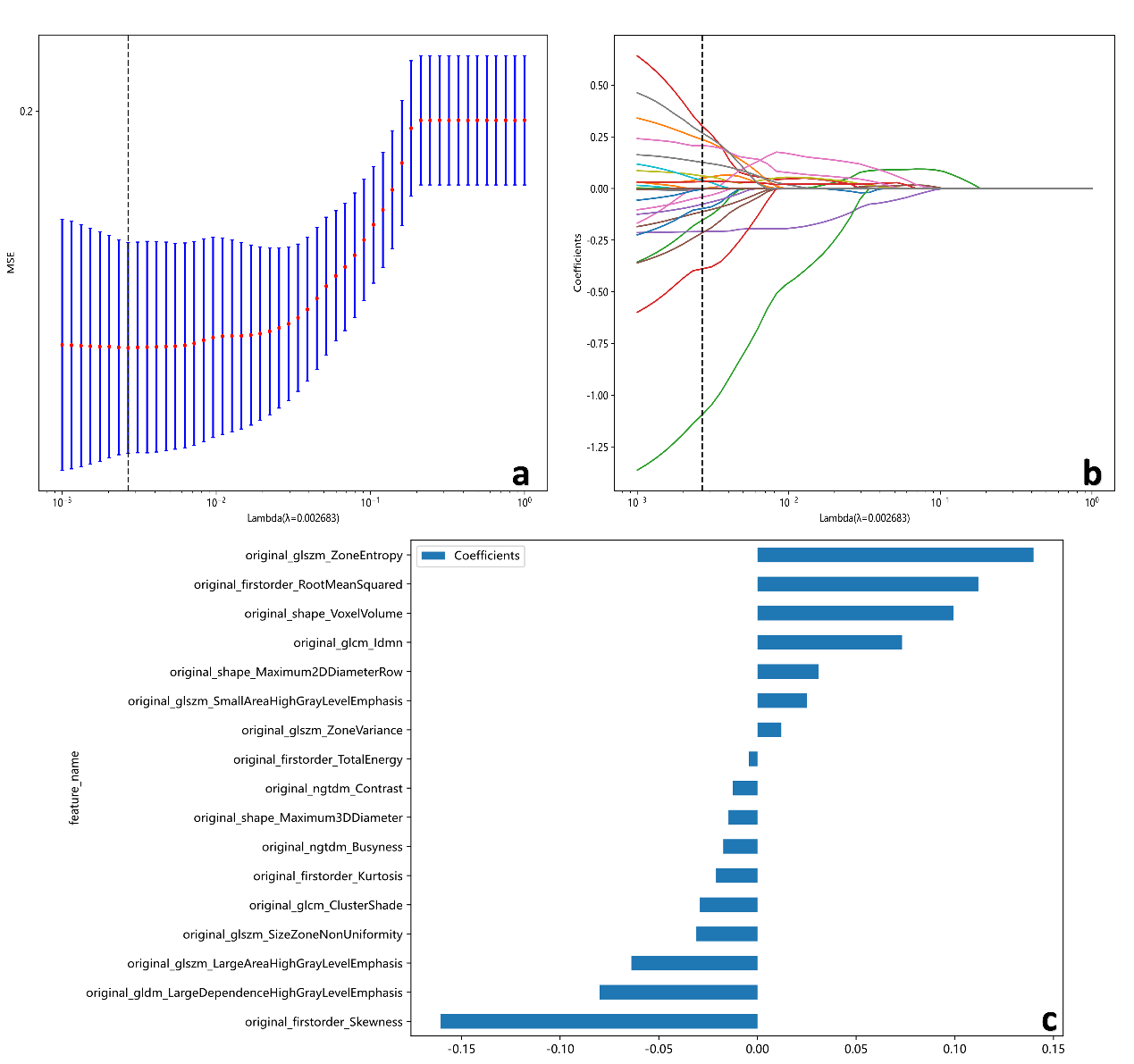
**

**Figure S2.** LASSO regression analysis procedure for the selection of radiomic features. (a) The λ parameter in the LASSO model was adjusted using 10-fold cross-validation to achieve the minimum mean square error. The optimal λ value is 0.0268, as indicated by the vertical dashed line. (b) Representative LASSO coefficient distribution plots. A vertical dashed line is drawn at the value selected after 10-fold cross-validation, based on the coefficient distribution map generated from the λ sequence. The best λ value was used to filter the non-zero coefficients. (c) Coefficient values for 17 features. LASSO, least absolute shrinkage and selection operator.


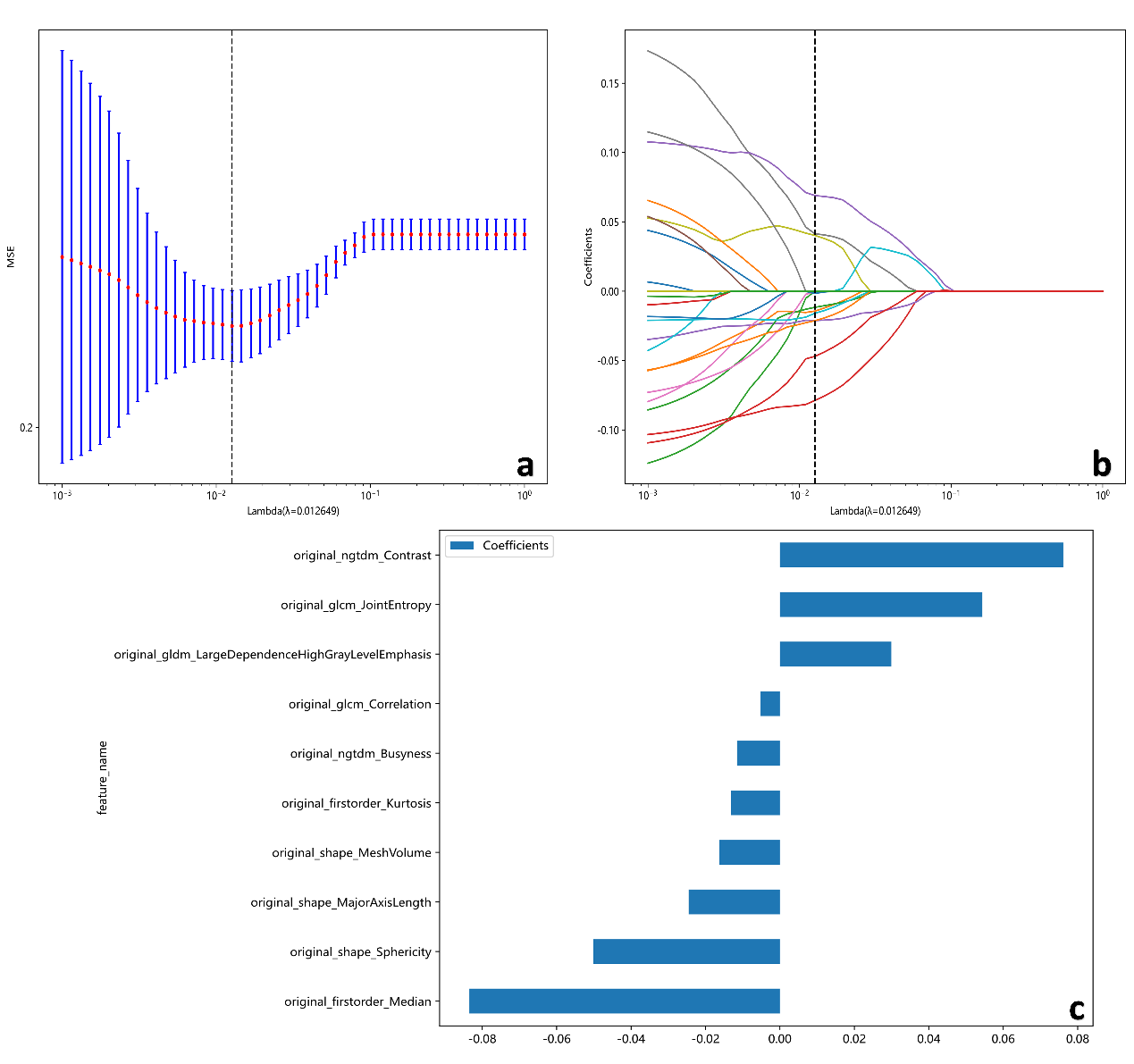


**Figure S3.** LASSO regression analysis procedure for the selection of radiomic features. (a) The λ parameter in the LASSO model was adjusted using 10-fold cross-validation to achieve the minimum mean square error. The optimal λ value is 0.0126, as indicated by the vertical dashed line. (b) Representative LASSO coefficient distribution plots. A vertical dashed line is drawn at the value selected after 10-fold cross-validation, based on the coefficient distribution map generated from the λ sequence. The best λ value was used to filter the non-zero coefficients. (c) Coefficient values for 10 features. LASSO, least absolute shrinkage and selection operator.


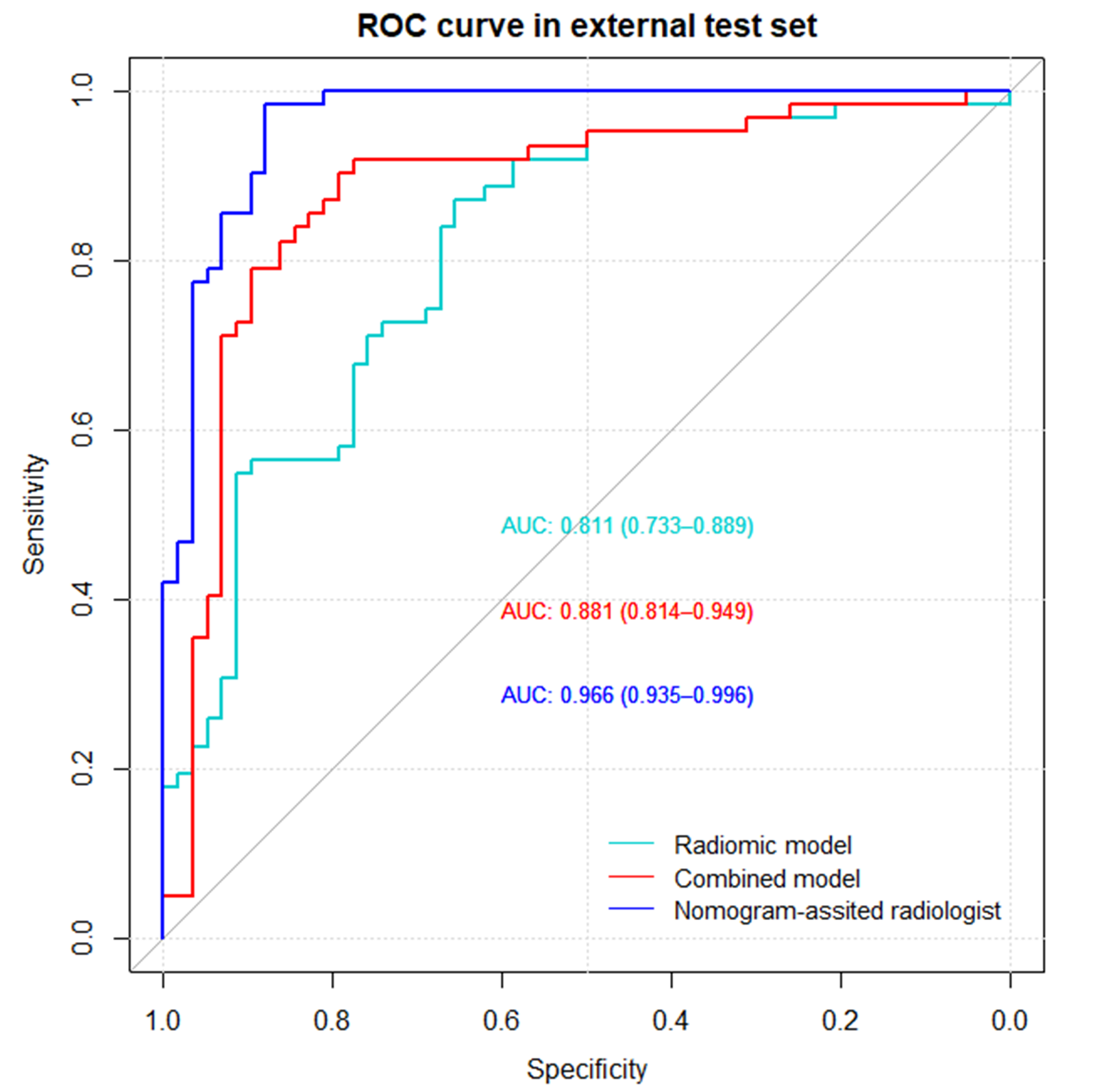


**Figure S4.** Receiver operating characteristic (ROC) comparison among radiomic model, combined model, and nomogram-assisted radiologist using the external test set.
